# Muscle weakness and lack of reflex gain adaptation predominate during post-stroke posture control of the wrist

**DOI:** 10.1186/1743-0003-6-29

**Published:** 2009-07-23

**Authors:** Carel GM Meskers, Alfred C Schouten, Jurriaan H de Groot, Erwin de Vlugt, Bob JJ van Hilten, Frans CT van der Helm, Hans JH Arendzen

**Affiliations:** 1 Department of Rehabilitation Medicine, Leiden University Medical Center, Albinusdreef 2, 2333 AL, Leiden, The Netherlands; 2 Department of Biomechanical Engineering, Faculty of Mechanical Engineering, Delft University of Technology, Mekelweg 2, 2628 CD, Delft, The Netherlands; 3 Department of Neurology, Leiden University Medical Center, Albinusdreef 2, 2333 AL, Leiden, The Netherlands

## Abstract

**Background:**

Instead of hyper-reflexia as sole paradigm, post-stroke movement disorders are currently considered the result of a complex interplay between neuronal and muscular properties, modified by level of activity. We used a closed loop system identification technique to quantify individual contributors to wrist joint stiffness during an active posture task.

**Methods:**

Continuous random torque perturbations applied to the wrist joint by a haptic manipulator had to be resisted maximally. Reflex provoking conditions were applied i.e. additional viscous loads and reduced perturbation signal bandwidth. Linear system identification and neuromuscular modeling were used to separate joint stiffness into the intrinsic resistance of the muscles including co-contraction and the reflex mediated contribution.

**Results:**

Compared to an age and sex matched control group, patients showed an overall 50% drop in intrinsic elasticity while their reflexive contribution did not respond to provoking conditions. Patients showed an increased mechanical stability compared to control subjects.

**Conclusion:**

Post stroke, we found active posture tasking to be dominated by: 1) muscle weakness and 2) lack of reflex adaptation. This adds to existing doubts on reflex blocking therapy as the sole paradigm to improve active task performance and draws attention to muscle strength and power recovery and the role of the inability to modulate reflexes in post stroke movement disorders.

## Background

Movement disorders after stroke are the result of a highly complex interplay between neuronal, muscular, and connective tissue characteristics, which is not yet fully understood. Evolving from Lance's concept of spasticity[1], a direct causative relation was assumed between hyperreflexia, muscle hypertonia/contracture and subsequent movement disorders. However, in a recent review paper, Dietz and Sinkjaer underline the discrepancy between clinically measured spasticity and functional spastic movement disorders and a more complex picture is sketched [2]. Next to altered reflex behaviour, changed visco-elastic properties of muscles and connective tissue [3-7] and the role of (impaired) voluntary muscle activation [8,9] are considered important factors. Furthermore, factors are interrelated, e.g. muscle mechanics will influence stretch reflexes [10] while changed muscle visco-elastic properties may be compensatory for the nervous system dysfunction [2,11]. Additionally, level of voluntary muscle activation will influence all aforementioned factors and interrelations [9]. It is therefore not surprising that it is still difficult to predict which patients will benefit from antispastic treatment [12,13].

In order to improve treatment strategies, it is important to quantify the role of both neuronal and muscular contributions to the movement impairment. Mainly because of the aforementioned interplay, this is faced with difficulties. Neuronal and muscular factors are to be separated by other means than differentiating in movement speed, as both factors are able to generate velocity dependent joint stiffness, i.e. viscous muscle properties and velocity sensitive stretch reflexes. Furthermore, measurements should be task defined, as passive measurements are likely not related to the active system state [2,13]. The application of external perturbation signals and subsequent closed loop system identification is a powerful tool to assess systems with an intact peripheral reflex feedback loop, as is the case in stroke [14]. A haptic manipulator was developed by which continuous torque perturbations can be applied to the wrist [15]. The subject holding the handle is to resist the resulting angular deviations maximally. These deviations are kept small to allow for a linear approach. Force/torque perturbations feel natural to the subject and trigger all available mechanisms to generate endpoint joint stiffness under maximal performance [16]. The used manipulator is controlled to respond to the mechanical behaviour of the subject attached, while the manipulator's apparent characteristics can be set. Thus, the mechanical load to the neuromusculoskeletal system can be manipulated [17]. The relationship between handle torque (input) and resulting angular deviation (output) yields the mechanical behaviour of the subject attached and comprises intrinsic muscle resistance and reflex mediated stiffness. Neuromuscular modelling is subsequently applied to identify the muscle visco-elasticity i.e. intrinsic resistance including muscle co-contraction and the reflex mediated resistance, i.e. the reflexive feedback loop gain.

The goal of this study was to quantify both factors in a cohort of patients with clinically diagnosed spasticity after stroke using aforementioned method. In order to study reflex modulation, two reflex provocative experiments were performed: 1) applying additional viscous manipulator loads; 2) reducing the perturbation signal bandwidth.

## Methods

### Patients and subjects

A convenience sample of n = 13 patients with a spastic paresis after stroke was recruited from the outpatient clinics of the Rijnland's Rehabilitation Center, Leiden, The Netherlands and the Leiden University Medical Center. A control group was composed of age, sex and arm dominance matched healthy subjects (Table 1). All patients had a Modified Ashworth Score [18] of ≥ 2, a functional disability (Brunstrom stage between 2 and 5 [19]) and enhanced tendon reflexes of either the mm. flexor and extensor carpi ulnaris or radialis on the affected side compared to the ipsilateral side. All subjects gave their written informed consent to the experiment, which was approved by the Medical Ethical Committee of the Leiden University Medical Center.

**Table 1:** Demographic characteristics & disease history

**Patient ID**	**Age (yrs)**	**Sex**	**Follow up (months)**	**Paretic side**	**Dominant side**	**Ashworth**	**Brunnstrom stage**
1	50	M	4	R	R	2	5
2	52	F	32	L	R	5	3
3	67	F	4	L	R	3	4
4	64	M	8	L	L	2	5
5	62	M	5	R	R	2	5
6	76	M	7	L	R	3	3
7	56	M	6	L	R	4	3
8	50	M	11	L	R	4	2
9	74	M	58	L	R	4	2
10	54	F	4	R	R	2	5
11	48	F	11	L	R	4	2
12	64	F	312	L	R	5	2
13	42	F	168	L	L	3	2

**Patients**	**58.4 ± 10.4**	**7**♂**6**♀	**48.5 ± 91.2**	**3R 10**	**11R 2L**	**3.31 ± 1.11**	**3.31 ± 1.32**
**Controls**	**58.1 ± 8.6**	**7**♂**6**♀	**-**	**-**	**11R 2L**	**-**	**-**

### Apparatus

**Figure 1:**
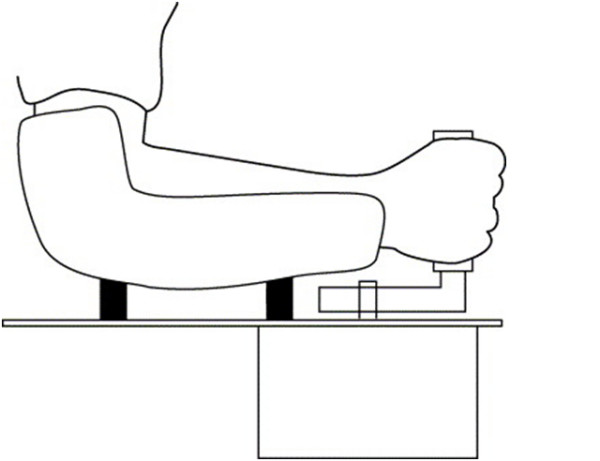
**The haptic manipulator**. Schematic drawing of the haptic manipulator. The subject is holding a handle, which is connected via a lever to the axis of an electrical motor, which is mounted underneath the surface of a table. The lower arm is fixated to the table.

A custom built haptic manipulator was used [15], consisting of a computer controlled electrical disc motor (Baumuller DSM 130N, Nürnberg, Germany) with a vertically oriented handle attached to the motor axis via a lever (Figure 1). The length of the lever was such that on average, when holding the handle, the rotation axis of the wrist coincided with the axis of the motor. Between the handle and the lever, a force transducer was mounted. The motor was equipped with an encoder to measure the (joint) angle (Stegmann SRS50, Düsseldorf, Germany).

A haptic controller was used which replaced the real manipulator dynamics with virtual dynamics, in this case a (rotational) visco-elastic load with inertia. This permitted: 1) estimation of the mechanical characteristics of the subjects from the mechanical behaviour of the total system, viz. man and machine, as subjects adapt to the load [17]; 2) adjustment of the loading conditions, i.e. inertia (*I*_*e*_), viscosity (*b*_*e*_) and elasticity (*K*_*e*_).

The motor was mounted underneath a table, on which surface adjustable clamps were mounted to fixate the lower arm. The subjects were seated while the arm/shoulder was positioned in about 45° internal rotation with respect to the frontal plane, with the elbow in about 90° flexion.

### Procedure

**Figure 2:**
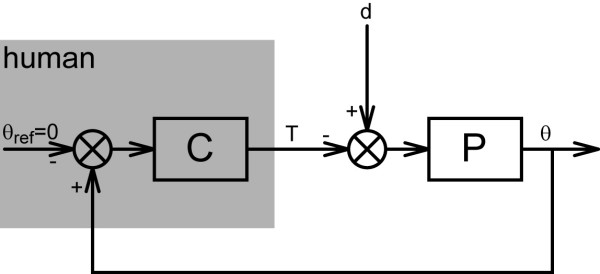
**Block scheme of the experimental set- up**. General scheme of the interaction between a subject and a haptic manipulator. The haptic manipulator imposes a virtual, or external, environment *P*. *C *describes the human controller, i.e. impedance of the wrist (inverse of admittance). Torque disturbance, *d*, together with the human (reaction) torque, *T*, are the inputs of the external environment, resulting in angle *θ*. During postural control, the objective of the human subject (grey box) is to '*maintain position*' and the internal reference angle will be constant, or zero: *θ*_ref _= 0.

**Figure 3:**
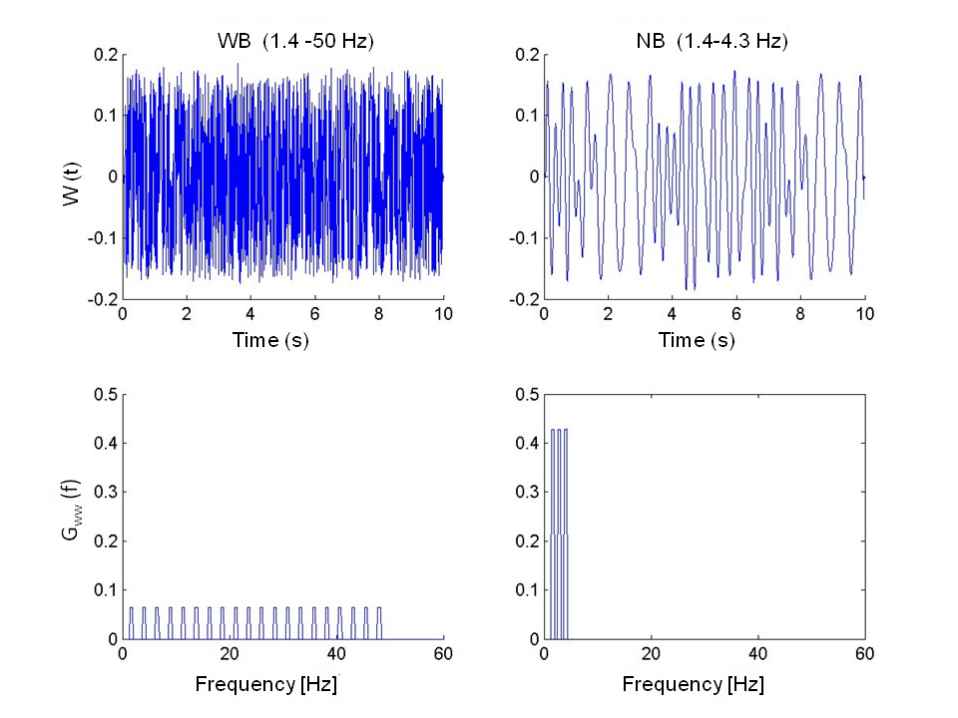
**Perturbation signals**. Examples of the perturbation signals; left: Wide Bandwidth (WB); right: Narrow Bandwidth (NB). Upper plots: time domain; lower plots: spectral densities of the signals. For the NB, one of the six applied signal bandwidths is shown. As the power of the perturbation signals was normalized per subject, units on the y-axis are dimensionless.

Subjects were asked to minimize displacements of the wrist while continuous random torque disturbances were applied to the handle of the manipulator (Figure 2). Displacements of the handle were shown on a computer screen to motivate the subjects and to control for angular drift of the handle from the neutral position. Perturbations were imposed upon the subject during trials of 10 seconds duration. Between each trial, a 5 second rest period was inserted to avoid fatigue. The perturbation signals were off line generated and delivered twice for each condition. Basically, two types of experiments were performed, comprising different types of perturbations (Figure 3):

1) Wide Bandwidth perturbations (WB): a perturbation signal with uniform power between 1.4 and 50 Hz [20,21]. Loading characteristics, i.e. *I*_*e*_, *b*_*e *_and *K*_*e *_were set to 1.6 gm^2^, 0 Nms/rad and 0 Nm/rad respectively. This condition is referred to as the reference condition. Additionally, viscous loads were applied, i.e. b_e _of 0.25, 0.50, 1 and 2 Nms/rad. The viscosity increases the stability margins and allows for increased reflex gains without the penalty of oscillations and thus, increased reflex activity is expected.

2) Narrow Bandwidth perturbations (NB) between a fixed bottom frequency of 1.4 and a variable upper frequency of 3.1, 4.3, 6.7, 9.1,11.6 and 16.5 Hz respectively. The loading characteristics *I*_*e*_, *b*_*e *_and *K*_*e *_were equal to the reference condition. Decreasing the frequency content of the signal and shifting signal power towards the lower frequencies will remove power from the natural oscillatory frequency of the wrist and thereby enhanced reflex gains are permitted to improve performance at the lower frequencies.

Thus, in total 22 trials were presented. The order was randomized in order to avoid anticipation.

### Data processing

#### Signal recording and basic processing

The recorded signals, viz. the motor (wrist joint) angle *θ(t)*, torque applied to the handle *T(t) *and the original perturbation signal *d(t) *were digitally recorded with a sample frequency of 2.5 kHz and a resolution of 16 bits. Electromyograms (EMG) of the wrist flexors and extensor muscles were recorded using two bipolar electrodes, placed in the middle of each muscle belly of the mm. flexor and extensor carpi radialis. Inter electrode distance was 20 mm. EMG signals were amplified, band pass filtered (20–1000 Hz), AD converted (16 bit resolution, sample frequency 2500 Hz) and rectified. EMGs of the FCR and the ECR were summed, were the FCR was positive and the ECR negative, as they operate in opposite direction. For each condition, the signals were averaged over the two repetitions. Data were processed using MATLAB version 7.04 (The Mathworks, Natick, Massachusetts, USA).

#### Non-parametric analysis

All signals were converted to the frequency domain using Fast Fourier Transformation. Frequency Response Functions (FRFs) were calculated by dividing the appropriate spectral densities as an estimate of the joint admittance : ratio of angle and torque per frequency. Admittance is the inverse of the impedance, i.e. the resistance of the wrist to applied torque:

(1)

Joint inertia, passive muscle visco-elastic properties including muscle (co-) contraction and spinal reflexes all contribute to the joint admittance.

Next to the mechanical admittance, the reflexive impedance was estimated:

(2)

The reflexive impedance was described by the summed flexor and extensor EMG activity as a result of the position deviations. As the gain of EMG is ambiguous, only the phase of the estimated reflexive impedance was used, which is affected primarily by the neural time delay of the reflexes.

Along with the FRFs, the coherences for the angle  were estimated. The coherence varies between 0 and 1 where a value of 1 indicates that the relation between input (perturbation or joint angle) and output signal (angle of motor/joint or EMG activity) is linear and free of noise.

#### Parametric analysis: neuromuscular modelling

**Figure 4:**
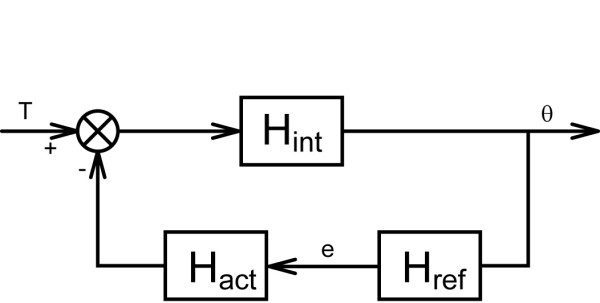
**Block scheme of the human controller**. Block scheme of the wrist admittance, representing (the inverse of) "C" in Figure 4. The wrist dynamics are the result of the interaction between the intrinsic dynamics *H*_*int*_, reflexive dynamics *H*_*ref *_and activation dynamics *H*_*act*_. The visco-elasticity as a result from co-contraction is included in H_int_, together with the wrist inertia. Angular deviations (θ) are sensed by the reflexive system (H_ref_) and result in a corrective torque. The imposed torque (T) together with the reflexive torque act upon the intrinsic system (H_int_) which result in the angular deviations.

To obtain physiological relevant parameters a neuromusculoskeletal model[22] was fitted on the mechanical admittance and the phase of the reflexive impedance simultaneously. The model incorporates wrist inertia (*I*), muscle viscosity (*b*), elasticity (*K*), neural time delay (*τ*_*d*_) and muscle activation dynamics. A complete model includes three reflex gains i.e. an acceleration, velocity and position dependent component (*k*_*a*_, *k*_*v*_, *k*_*p*_). For the wrist, we alternatively used a model including only the velocity dependent component i.e. *k*_*v*_. The muscle activation dynamics describe the muscle force built-up. The activation dynamics are modelled by a second order filter with fixed parameter settings, i.e. a bandwidth of 2.17 Hz and a relative damping of 0.75 [21] (Figure 4).

(3)

(4)

The model was fitted onto the measured mechanical admittance and phase of the reflexive impedance by minimizing the following criterion function:

(5)

where  and *H*_*ref*_ ( *f *) have a normalized amplitude of one. Only frequencies where the perturbation signal contained power were included. Because of the large range of the FRF gain, a least squares criterion with logarithmic difference was used [23]. The criterion was weighted with the coherence to reduce emphasis on less reliable frequencies in the FRF and with (1+f)^-1 ^to prevent excessive emphasis on the higher frequencies [21].

The express the 'goodness' of the fit, the Variance-Accounted-For (VAF) was calculated [21]. To calculate the VAF, simulated and recorded angle were compared. A VAF of 100% indicates that the model fully predicts the measured angle. The VAF is reduced by signal noise and other unmodelled behaviour.

### Stability analysis

The mechanical (in) stability, i.e. the tendency to oscillate was estimated by calculating the phase shift (phase margin) needed to reach instability of the total system of manipulator and subject [16,24].

### Statistical analysis

A repeated measurements General Linear Model ANOVA was used to test the effects of adding viscous loads (experiment 1) and changing the perturbation frequency bandwidth (experiment 2). Both viscous load and frequency bandwidth were modelled as within subject factors and control versus patient as between subject factor. A one-way ANOVA was used to compare the phase margins of patient versus controls. All tests were performed with an α of 0.05 using SPSS 11.5.

## Results

### Signal and model validity

Averaged over all excited frequencies, signal coherence for the patient group was 0.87 SD 0.15 during the reference condition and above 0.95 for the viscous loading conditions. For the control group, coherence was above 0.99 for all conditions. Variance Accounted For (VAF) for the reference condition for the patient group was 75 SD 14% versus 84 SD 6.3% for the control group. VAF for the viscous loading conditions was above 91% for both patient and control group. Averaged over all conditions of the reduced bandwidth perturbations, VAFs for the patient and control group were 74 SD 17% and 78 SD 12%. Combining a position feedback gain k_p _with k_v _in the reflexive model resulted in a substantial lower VAF, i.e. 56 SD 28% and 83 SD 11% averaged over reference condition and viscous loading conditions for the patient and control group respectively. Further results of the study are based upon a model including k_v _only.

### Experiment 1: increasing the viscous loading

**Figure 5:**
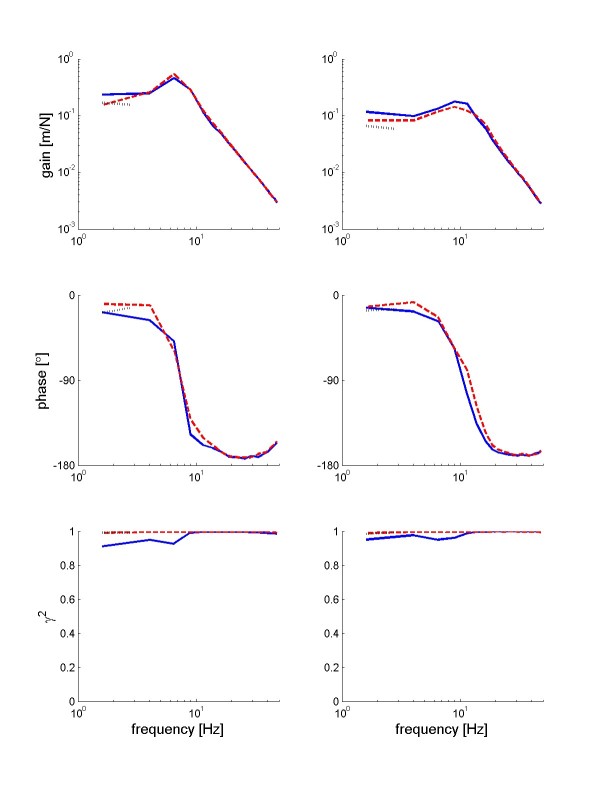
**Example of FRFs**. Typical examples of FRFs of a stroke patient (left) and a control subject (right) for the mechanical admittance together with corresponding coherences. Upper row: gain, the solid blue line represents the reference condition, the red dotted line the WB disturbance with a viscous load of 2 Nms/rad and the dashed black line a NB disturbance (1.4–4.3 Hz). Middle row: phase; bottom row: coherences.

Figure 5 shows typical examples of the FRF of a patient and a control subject respectively. The patient's wrist admittance was higher compared to the control subject. In both cases, the admittance increased at higher frequencies, due to a tendency to oscillate at the eigen frequency of the wrist (about 10 Hz). With increased damping of the environment, the wrist admittance decreased, implicating a stiffer joint. The phase lag with increasing frequency resulted from the neural time delay.

**Figure 6:**
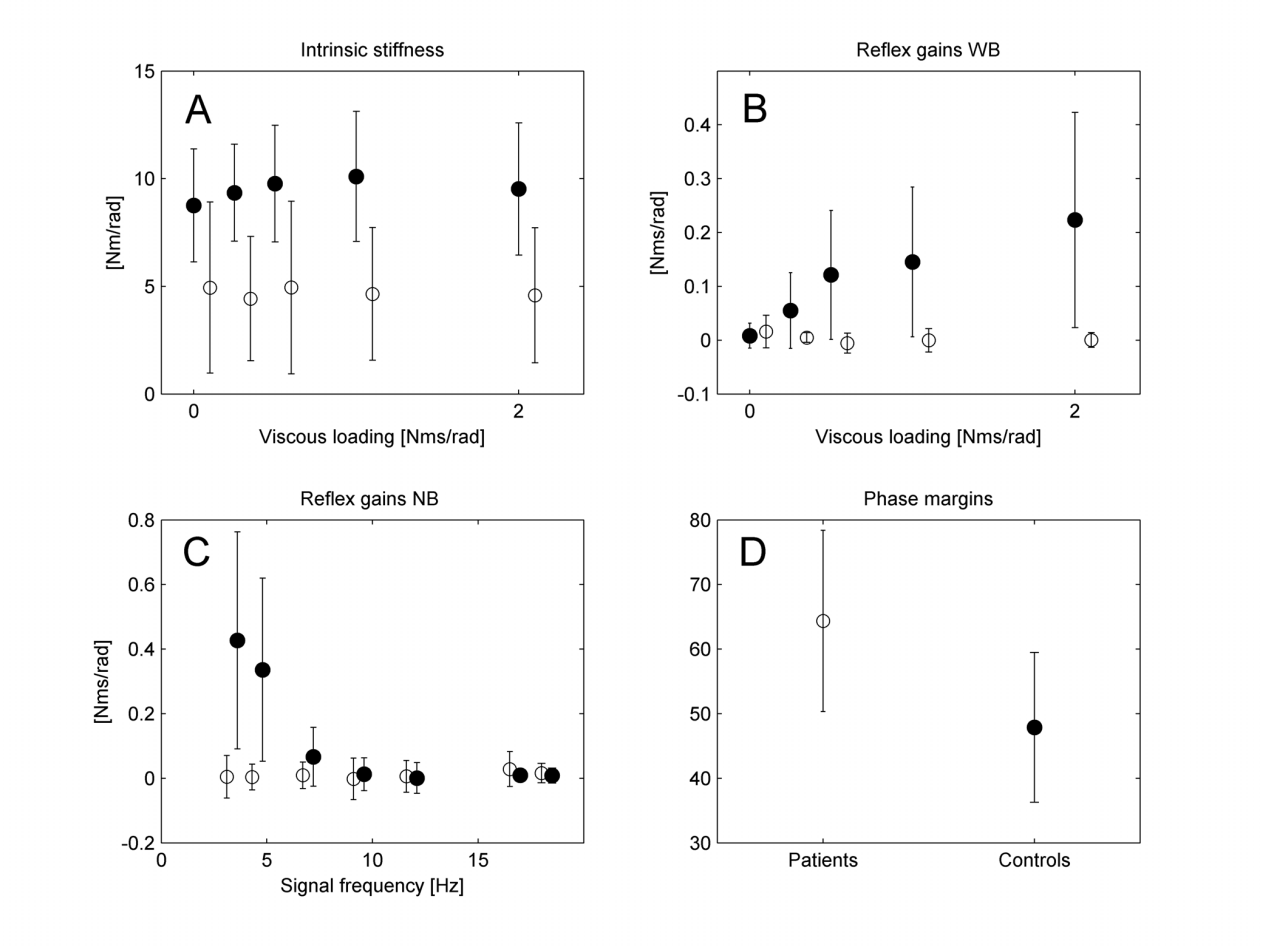
**Main outcome parameters**. Panel A: intrinsic stiffness (elasticity) including muscular co-contraction as a function of increasing viscous load during WB perturbations for controls (black circle) and patients (open circle) respectively. The error bars represent the group standard deviations; panel B: reflex gains (*kv*); panel C: reflex gains during NB perturbations for controls (black circle) and patients (open circle) respectively. The last 2 error bars (patients and controls) represent the reference condition (WB perturbations, no viscous loading); Panel D: results of stability analysis: phase margins for controls(black circle) and patients (open circle) respectively.

On parameter level, muscle elasticity of the patient group was significantly smaller than the control group: mean over the reference condition and viscous loading conditions: 4.71 SD 3.32 vs. 9.50 SD 2.66 Nm, between subject effect p < 0.001, Figure 6a.

Viscous loading caused a significant increase of the reflex gain *k*_*v *_in the control group, while this was not the case in the patient group: within subjects effect p < 0.001, interaction term p < 0.001, Figure 6b. During the reference condition, the reflex gains of patients were comparable to healthy subjects. During viscous loading, the reflex gains in the control group were tuned up while this was not the case in the patient group. There were no significant differences between control and patient group concerning the other parameters except for the phase margins which were significantly larger in the patient group: 64 SD 14 vs. 46 SD 13°, p = 0.014, Figure 6d. The phase margins were only evaluated for the reference condition.

### Experiment 2: varying the perturbation bandwidth

The results of this experiment are shown in Figure 6c. As can be observed, reducing the perturbation bandwidth led to increased *k*_*v *_for the control group, while this was not the case for the patient group (within subjects effect p < 0.001 with interaction term p < 0.001). As such, *k*_*v *_modulated with perturbation bandwidth in the normal case while this modulation was lost in the patient group.

## Discussion

We used a novel approach to estimate different contributors to joint (wrist) stiffness of patients post stroke during an active posture task. The method makes use of a haptic manipulator to deliver torque perturbations and subsequent linear closed loop system identification and neuromuscular modelling to analyze and express joint admittance into relevant parameters. Intrinsic stiffness of patients post stroke was about 50% of healthy subjects. Reflex contribution was found not to respond to reflex provoking conditions in contrast to control subjects. Patients were mechanically more stable than control subjects.

### Validity of the experimental approach

The high signal coherences justify the linear approach used in this study. The VAF values indicate that the measurements can be well described by the used model. The VAF decrease when including the position feedback gain justified the use of only one, velocity dependent reflexive feedback gain into the model. This assumption can be theoretically underpinned: 1) considering the relatively high eigenfrequency of the wrist, in contrast to e.g. the shoulder joint, the role of reflexive position feedback is relatively small compared to velocity feedback; 2) the perturbation frequencies were 1.5 Hz and higher, while reflexive position feedback will have the largest contribution below 1.5 Hz.

### Hyperreflexia or not?

Although reflex gains for patients and controls were comparable during the reference condition, we found no evidence of functionally enhanced reflex gains in patients. Enhanced reflex gains relative to the intrinsic characteristics i.e. muscle visco-elasticity would drive the system to instability i.e. oscillatory behaviour. By calculating the phase margins as a parameter of system stability we found that patients were actually more stable than controls. Explained from optimal control theory, healthy subjects are apparently capable of tuning their reflexive gains to increase performance at the cost of smaller stability margins. Patients remain on the safe side and/or are not capable of this reflex tuning.

### Role of impaired reflex modulation

Only a few studies so far have addressed the issue of reflex modulation in stroke. Impairment of reflex modulation was found previously during walking [25-27].

We addressed the upper limb i.e. the wrist during maintenance of posture and found patients not to adjust reflex gains to provoking conditions. During posture maintenance and reactive to changing loading conditions, constantly a balance is sought between energy efficient reflex stiffness and energy costly co-contraction [17]. In reaching this balance, the modulation of reflex gains is a key factor. Model studies showed that normally this modulation is optimal to improve performance [17,28]. While subjects seek to be close to instability and thus are making optimal use of their reflexes, patients do not. Thus, theoretically, optimal control of reflexes is very important, however, the precise mechanisms as well as the implications for function need further elaboration. Besides the optimal control viewpoint which implies instantaneous supraspinal tuning of reflex gains, other mechanisms of reflex modulation are imaginable. E.g. by increasing the viscous loading or reducing the perturbation bandwidth, the velocity content of the signals is lowered, which may induce reflex activity at the level of the reflex loop itself (spindle dynamics or neurotransmitter release).

### Passive vs. active measurements

The main difference between the current experiment and common clinical testing is the fact that we measured under active conditions. Under these circumstances, the paresis component will become evident. This revealed itself by the 50% drop in intrinsic stiffness, which is the result of passive viscoleastic properties, modified by muscle co-contraction. Muscle weakness dominating over hypertonia during voluntary movement was found previously [29-32]. The low intrinsic muscle stiffness found in a number of patients indicates that enhanced passive joint stiffness which is so evident under passive testing conditions is masked under active conditions. Mirbagheri et al. [33] found in studies addressing the ankle and elbow joint, a high intrinsic stiffness and high reflex gains under passive conditions using position perturbations.

The absence of enhanced reflex activity under active conditions confirms previous findings [34-38]. According to Burne et al [39], spasticity may be fully explained by the inability of patients to tune their reflex activity down together with the active muscle contraction state, thus still exhibiting relatively high reflexes under normally relaxed conditions. Although the absence of reflex modulation under provoking conditions and the domination of low intrinsic muscle stiffness in patients suggest that spasticity is a more complex disorder, again the importance of test conditions on the outcome is underpinned [40].

### Posture vs. movement

In order to allow for a linear approach, the positional deviations in the present study were kept small. It appeared that including only a velocity dependent reflexive feedback gain into the reflexive model was sufficient for a good fit of the experimental data and no position reflexive feedback was required to explain the dynamics.

Commonly, in assessment methods the joint is moved over considerable trajectories. This will not only have profound impact on the behaviour of the intrinsic visco-elastic properties of muscles and connective tissue and but also on the reflexive feedback system. Three mechanisms may be responsible for impaired reflex activity as a function of motion trajectory. First, it is suggested that the II or Ib afferents (respectively spindle position and Golgi tendon organ) are hyperactive instead of the Ia afferents (spindle velocity) [11]. This means that hyperreflexia would be revealed only during significant length changes and forces of muscles. Secondly, impaired adaptation of reflexes, e.g. due to lack in task-dependent modulation of Renshaw cell activity [41,42] may lead to improper adjustment to muscle length changes and thereby to untimely joint resistance [26,27]. Thirdly, disynaptic reciprocal Ia inhibition may become evident during significant joint motion [43].

Non-linear system identification approaches are required for non-linear conditions i.e. moving a joint over a considerable trajectory. These techniques are currently being developed by our group.

We measured in a single neutral joint position. It may be that different characteristics are found in wrist flexion or extension as evidence was found for operating point dependency in spasticity [44-46].

### Clinical implications

The results of the present study further underline the discrepancies between outcome during passive and active assessment. In judging post stroke movement disorders, the information of based on clinical tests performed under passive conditions should be applied with caution, as mechanical behaviour may be completely different. This was also demonstrated for balance and standing in stroke [47].

Therapeutically, at least for improving posture and balance, reflex blocking therapy seems less appropriate, considering the reduced reflexive feedback we found during active tasks, while further decline of muscular strength may be very counterproductive. Instead, enhancement of muscle strength is required. Strengthening exercises as a part of rehabilitation programs was found to be beneficial [48]. Further research is required to investigate whether return of muscle strength goes with return of reflex modulation ability, or when this is not the case what the actual limiting factor is for regaining functionality.

### Limitations

It should be noted that the measured cohort of patients was small and possibly did not cover the entire clinical spectrum. A single joint approach does not allow for studying inter limb coordination and the influence of body posture on reflex characteristics, i.e. postural reflexes. These issues will be covered in future work.

## Abbreviations

*Ie*: Manipulator inertia [gm2]; *be*: Manipulator viscosity [Nms/rad]; *Ke*: Manipulator stiffness [Nm/rad]; *I*: Inertia of the wrist [gm2]; *b*: Intrinsic viscosity; including muscle (co-) contraction [Nms/rad]; *K*: Intrinsic elasticity (or stiffness); including muscle (co-) contraction [Nm/rad]; *kv*: Velocity feedback gain [Nms/rad]; *kp*: Position feedback gain [Nm/rad]; *θ(t)*: Joint angle [degrees]; *T(t)*: Joint torque [Nm]; *d(t)*: Perturbation signal (a series of random torque perturbations) [Nm]; FRF: Frequency Response Function; describes the dynamic relationship between torque and angle; i.e. ratio per frequency in the frequency domain; : Coherence as a measure of the linearity of the angle:torque relationship. The coherence varies between 0 and 1; a value of 1 means that at a specific frequency the relation between input and output is linear and free of noise; VAF: Variance Accounted For. Regarding the angular position, calculated as the ratio of the simulated angle to the actual measured angle, expressed as a percentage.

## Competing interests

The authors declare that they have no competing interests.

## Authors' contributions

CGM carried out the measurements, performed the statistical analysis and prepared the manuscript. ACS designed the experiment, wrote the data processing software, performed the data processing and edited the manuscript. JDG assisted in data interpretation and commented on the manuscript. EDV assisted in experimental design and writing of data processing software. JVH assisted in data interpretation and commented on the manuscript. FVH conceived of the study, assisted in data interpretation and commented on the manuscript. JHA assisted in data interpretation and commented on the manuscript. All authors read and approved of the manuscript.
